# Assessment of Renewable Energy Technology and a Case of Sustainable Energy in Mobile Telecommunication Sector

**DOI:** 10.1155/2014/947281

**Published:** 2014-01-23

**Authors:** Michael S. Okundamiya, Joy O. Emagbetere, Emmanuel A. Ogujor

**Affiliations:** ^1^Department of Electrical and Electronic Engineering, Ambrose Alli University, Ekpoma P. M. B. 14, Nigeria; ^2^Department of Electrical and Electronic Engineering, University of Benin, Benin City P. M. B. 1111, Nigeria

## Abstract

The rapid growth of the mobile telecommunication sectors of many emerging countries creates a number of problems such as network congestion and poor service delivery for network operators. This results primarily from the lack of a reliable and cost-effective power solution within such regions. This study presents a comprehensive review of the underlying principles of the renewable energy technology (RET) with the objective of ensuring a reliable and cost-effective energy solution for a sustainable development in the emerging world. The grid-connected hybrid renewable energy system incorporating a power conversion and battery storage unit has been proposed based on the availability, dynamism, and technoeconomic viability of energy resources within the region. The proposed system's performance validation applied a simulation model developed in MATLAB, using a practical load data for different locations with varying climatic conditions in Nigeria. Results indicate that, apart from being environmentally friendly, the increase in the overall energy throughput of about 4 kWh/$ of the proposed system would not only improve the quality of mobile services, by making the operations of GSM base stations more reliable and cost effective, but also better the living standards of the host communities.

## 1. Introduction

The significance of energy resources cannot be overemphasized, as they are essential in virtually all sectors of the economy. Electricity is the most widely used and desirable form of energy globally. It plays a substantial role in the socioeconomic and technological development of a country. In addition, the electricity demands of a country multiply with an increase in the population and economic development [1]. The rise in demand if not satisfactorily met can lead to a shortage in electricity supply, with adverse socioeconomic and environmental implications. Nigeria has been experiencing extreme electricity shortage characterized by high unreliability index for over two decades. This has made most entrepreneurs resort to the use of fossil fuelled generators either as supplements to the national grid or exclusively in remote sites.

The earth's climatic change is the result of increasing concentrations of greenhouse gases (GHGs) resulting primarily from fossil fuel combustion into the atmosphere. The environmental consequences of harnessing and utilizing the fossil fuels are assuming alarming proportions [2–11]. In addition, the global warming of the earth poses serious threats to the ecosystem and the most vulnerable is the coastal region. A large percentage of Nigeria's urban population lives in coastal cities. Moreover, most of the economic activities located within the coastal zone form the backbone of the national economies. Nigeria is already experiencing adverse impacts of climatic changes on agriculture, power generation, and tourism. A typical example is the recent flooding in the country, which claimed lives, displaced citizens, and destroyed properties (estimated over one billion dollar). This could severely affect almost all sectors negatively if unmitigated. Consequently, there arose a clarion call for a sustainable energy solution.

The goal is to attain negligible anthropogenic emissions of carbon dioxide, which constitute by far the largest part of the emissions of GHGs, thereby making the environment much more friendly and safe [12]. This has increased interest in energy saving and environmental protection achievable through the reduction in energy consumption and the extensive utilization of renewable energy sources [13]. The RET is capable of supplying the energy needed for rapid development. Solar and wind are commonly used renewable energy sources in recent years, on the basis of their environmentally friendly nature. The hybrid system, if optimally designed, can be more cost effective and reliable than a single-renewable energy system, and so there is increasing interest in determining the necessary conditions to install hybrid energy systems [14]. In addition, potential investors are at a cross road on the choice of the renewable energy mix and the optimum design specification for a given location, owing to the significant investment cost of the infrastructure and its dependence on climatic conditions.

This paper examines the RET, considering the prospects of resource availability and challenges for deployment of existing technologies in emerging economies using Nigeria as a case study. In addition, it presents a case for deploying a sustainable energy solution in mobile telecommunication sites at present, and in the future by making projections based on the assumption of future population and mobile infrastructure growth rates. Simulation studies performed under different climatic conditions, using practical load profile of a GSM outdoor base station, are presented, discussed, and compared with the present scenario. This is part of research efforts in solving the current electricity related problems of mobile telecommunications sector of developing countries.

## 2. Energy Conversion and Storage Systems

The Renewable energy system design usually integrates renewable energy mix, such as biomass, wind, and solar energy. Inauspiciously, large area of land, water usage, and social impacts often characterize the electricity production from biomass, and this requires further study to verify the technoeconomic viability of its power generation [17]. Consequently, it may be required to shift demand to other energy sources, such as wind and solar. Wind and solar energy are ubiquitous and freely available. They are used sources for renewable energy generation because they are both technically and environmentally viable options.

### 2.1. Wind Energy Conversion System

Wind energy is one of the most viable and promising sources of renewable energy globally. Accurate estimate of wind speed distribution, selection of wind turbines, and the operational strategy and management of the wind turbines are essential factors that affect the wind energy potential. One of the first steps a utility company considers when deploying wind as an energy source is to examine the available wind speed [12]. The next step is to adjust the wind speed data at anemometer height to wind turbine hub height using appropriate conversion ratio. The adjustment of the wind profile is necessary to account for the effects of the wind shear inputs. Moreover, accurate assessment of wind power potential at a site requires detailed knowledge of the wind speeds at different heights [18, 19]. Methods are available in the literature for improving the estimate of the hub height wind resource [20].

Hameed et al. [21] presented an overview of the techniques, methodologies, and optimization algorithms developed for monitoring the performance of wind turbine generators (WTGs) and early error detection to avoid catastrophic conditions caused by sudden breakdowns. Some authors have centred their interest on optimizing the turbine settings in order to maximize the performance. Jensen et al. [22] presented an overview of methods applied to the optimal design of wind turbine blades. Conversely, the determination of the optimal positions of the WTG is necessary in order to maximize the energy output. Kusiak and Song [23] proposed a multiobjective evolutionary algorithm for WTG placement based on wind distribution with the aim of maximizing the wind energy captured while minimizing an index, which determines constraint violation.

#### 2.1.1. WTG Model

Yang and Aydin [24] carried out a theoretical study on windmill and developed a revised model for power density and output power of a WTG. Malinga et al. [25] studied the dynamics and controlled the wind turbine as a distributed resource using the basic WTG design and a relationship for the power efficiency of the WTG proposed by Justus [26]. While [27, 28] assumed that the wind turbine power curves have linear and quadratic characteristics, respectively, [29–35] assumed a cubic characteristic and modelled the output power of a WTG according to the cube law. The power output of a turbine is a function of air density, swept area, and the cube of the wind speed. Altitude affects air density, which in turn affects the output of WTGs. Okundamiya and Nzeako [12] examined the effect of altitude on the output power of WTGs and proposed a robust model to account for this effect in the optimal sizing of a modern wind energy conversion system (WECS) based on the manufacturer's power profile. The study further determined the value of power law exponent for the estimation of wind speeds at different heights, if wind speed measurements are available at one height.

### 2.2. Photovoltaic Conversion System

The performance of the photovoltaic conversion system (PVCS) is highly dependent on its orientation and period of service [36, 37]. The orientation of the PV surface is described using its tilt angle and the azimuth, both relate to the horizontal. This creates the problem of designing the optimum tilt angle for harvesting solar energy at fixed latitudes, as this is essential for effective harnessing and utilization of global solar radiation.

Temperature affects crystalline cells and their performance decreases as cell temperature rises. A significant issue of concern is the heat build-up under the PV modules, resulting in the possible structural damage of the panel (if panels are unvented or if heat is unrecovered) and the lower efficiency of most PV modules with increasing temperature. This makes cooling required at high illumination conditions such as concentrated sunlight, or tropical conditions. The use of commercially available PV modules combined on top of the transpired collector and the utilization of the unique air balancing features of the transpired collector remove heat from the back of the PV array. This concept allows for heat transfer from most PV modules currently available in the market [38]. Removing the excess heat generated by the PV modules increases the electrical output.

In general, there are two steps in determining the available solar energy when supplying a remote load. The first step involves the determination of the amount of solar radiation that arrives on the earth at the PV panel's location. The next step is modelling of the panel itself, considering its efficiencies, losses, and physical orientation. Each step requires a model that deals with many variables, and inputs into the second stage of the model utilize the results of the first step. Global and diffuse solar radiation data are not measured at the forty-five (45) meteorological stations in Nigeria [39]. In the absence of these data, one has to rely on alternative methods of evaluation.

#### 2.2.1. Solar Radiation Models

There are several models proposed for the estimation of global- and diffuse-solar radiations. Liu and Jordan [40] developed a theoretical method for deriving the average hourly solar radiation from the average daily total radiation, with the assumption that the atmospheric transmission is constant throughout the day, and this is independent of solar altitude. Using the collected data for five U.S. stations and Liu and Jordan's curve, Collares-Pereira and Rabl [41] developed an analytical expression for the ratio of hourly to daily solar radiation, in terms of sunset hour angle, while [42, 43] developed regression models based on meteorological data for estimating the monthly average values of daily global solar radiation. Kaplanis [44] described a computationally efficient and intuitively basic model for the estimation of hourly global solar radiation on a horizontal surface. Sonmete et al. [45] examined and compared 147 available solar radiation models for the prediction of monthly solar radiation at Ankara (Turkey) based on selected statistical measures such as percentage error, mean percentage error, root mean square error, mean bias error, and correlation coefficient. Results showed that the Ball et al. [46] model and the Chen et al. [47] model performed best in the estimation of solar radiation on a horizontal surface for Ankara. Wan Nik et al. [48] determined the performance accuracy of selected monthly average hourly global solar radiation models [41, 44, 49–51]. Jakhrani et al. [52] examined the performance of four tilted surface solar radiation models [53–56] for the estimation of solar radiation on the basis of one-sample statistical test and concluded that the Klucher model [54] could be preferred for the estimation of tilted surface radiations in Kuching (Malaysia).

In Nigeria, several investigations [57, 58] have demonstrated the predictive ability of the Angstrom-type model [59], correlating the global solar radiation to relative sunshine duration in a linear regression form. Falayi et al. [60] based their studies on the correlation between global solar radiation and three meteorological parameters using monthly average daily data obtained from Iseyin, Nigeria. Okundamiya and Nzeako [61] proposed a temperature-based model for predicting the monthly average global solar radiation on horizontal surfaces for selected cities with varying meteorological conditions, representing the six geopolitical zones in Nigeria. Subsequent analysis [62] demonstrated the availability of diffuse solar radiation on a horizontal surface within the region employing clearance index.

#### 2.2.2. PV Model

Using the available solar radiation at the tilted PV surface, the air temperature, and manufacturers data for a PV module as input parameters, the power output of the PV module can be deduced [63]. The sizing optimization of PV system is a complex optimization problem aimed at achieving a cost-effective and efficient energy solution for the consumer.

Some authors have tackled the PV sizing problem using the tabu search [64] and conventional optimal search methods [32] and investigated the effects of model generated solar radiation data handling in hybrid sizing studies [33]. A constant PV generator efficiency was assumed in [32, 33] and as such neglected the effect of temperature on the PV module while [64] applied a derating factor to account for the effect of temperature on the PV system efficiency. The efficiency of the PVCS does not strongly depend on irradiance only, but also on the module temperature [65].

### 2.3. Hybrid Energy System

There has been outstanding interest in the optimal design and management of stand-alone hybrid energy systems with the aim of achieving energy balance between the maximum energy captured and consumed energy [66]. The fluctuating renewable energy supplies, load demands, and the nonlinear characteristics of some components complicate the design of hybrid systems. In addition, the overall assessment of autonomous hybrid energy systems that incorporate renewable and convectional energy sources depends on economic and environmental criteria, which are often conflicting objectives.

Among the various methods for sizing hybrid energy systems, the heuristic optimization tools, particularly genetic algorithms, and particle swarm optimization are frequently used probably for their simplicity and ability to produce satisfactory results [67]. The energy control strategy plays a vital role in the optimal design and efficient utilization of hybrid energy systems, as it affects the available power supply and the overall lifetime of the system components.

The technical constraints in hybrid energy systems relate to system reliability. Several reliability indices have been employed for the evaluation of generating systems in the literature. The most technical approaches used for the evaluation of power system reliability are the loss of load probability, loss of load power supply, and loss of power supply probability. Several other factors, which contribute to the expected profitability of renewable energy system, influence the economic viability. There are many ways to calculate the economic viability of distribution generation [68]. The choice of a model would depend on the sector for which the analysis is being performed. The technoeconomic analysis usually looks at the cheapest cost of energy produced by the system components while neglecting the excess capacity of the combination.

### 2.4. Energy Storage System

Power fluctuations could be incurred since the renewable energy sources are highly dependent on weather conditions. The use of batteries in medium and high power applications is not feasible [69]. Okundamiya and Nzeako [70] presented a detailed analysis of different battery models. The conclusion was that the use of batteries in supplying the instantaneous energy demand is inappropriate inasmuch as fluctuations negatively affect their lifetimes. A feasible option is the integration of super-capacitor (SC) as a part of the energy storage (backup) system to meet the instantaneous power requirement. SCs have several advantages including a remarkably high energy density as compared to conventional capacitors, long life cycle, temperature stability, no maintenance requiremnet, and environmental friendliness [71]. In addition, SCs can be charged and discharged continuously without degrading as batteries do. This makes them suitable candidates as energy storage devices in renewable energy applications. Supercapacitors will provide power to the system when there are surges (energy bursts) since supercapacitors can be charged and discharged immediately. In contrast, batteries can provide the bulk energy since they can store and deliver larger amount of energy over a longer and slower time.

The required energy storage capacity can be reduced to a minimum when there is optimal sizing of the energy system at a given site. A rate of prediction at which the energy storage unit charges (discharges) when the generated power is more (less) than the demanded power requires accurate energy storage model. Accuracy of the model in optimizing the lifetime and capacity of the energy storage unit is needed. The model should also account for protection against overvoltage and undervoltage (to prevent battery overcharge and overdischarge).

### 2.5. A Case of Sustainable Energy in Mobile Telecommunications Sector

**Figure 1:**
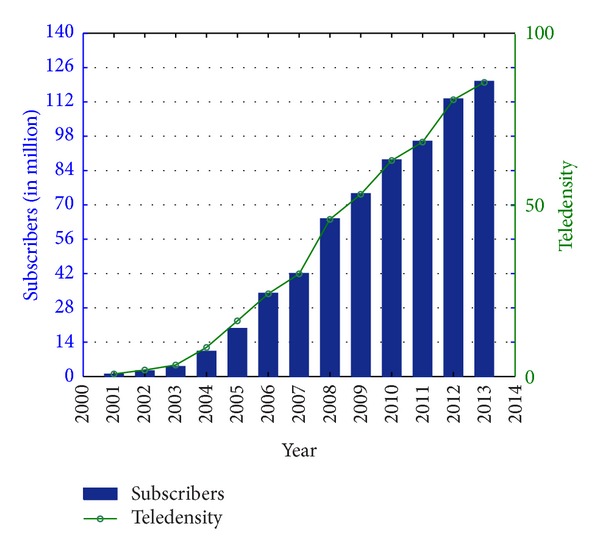
Trend of subscribers' base and teledensity in the period (2001–June 2013) in Nigeria [15].

**Figure 2:**
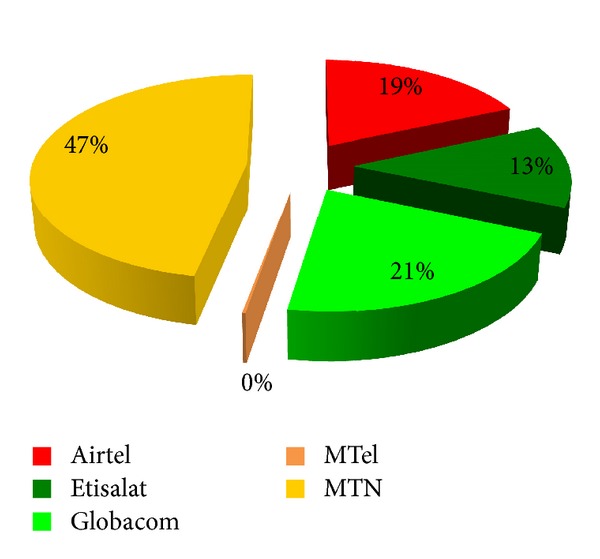
Market shares of GSM network operators as in June 2013 in Nigeria [15].

The mobile telecommunication sector is one of the fastest growing sectors of global economy. The ever-increasing demands of mobile telecommunication services in developing countries are driving this significant economic growth [72]. Nigeria currently has Africa's largest mobile telecommunication market with a teledensity of about 86 telephone lines per 100 people as in June 2013 [15, 72]. The growth of the Nigerian telecommunications sector since the inception of GSM in 2001 is unmatched by any other sector and it has recorded a phenomenal growth both in terms of subscribers' base and infrastructural development in the country.  Figure 1 shows the trend of subscribers' base and teledensity in the period (2001–June 2013) while Figure 2 shows the market shares of GSM network operators as in June 2013 in Nigeria [15].

The rapid growth of mobile telecommunications creates a number of problems such as network congestion and poor quality of service delivery. These problems are fast eroding the gains of the Nigerian mobile telecommunication sector [73]. Nevertheless, the lack of a reliable utility grid and the cost implication of a supplementary energy source are major problems besetting this sector in most developing countries, particularly as network operators strive to expand their communications network to provide global coverage with increased quality of service. Most mobile telecommunication sites in the developing world rely heavily on the use of fossil fuelled generators either as supplements to the utility grid or exclusively in remote locations.

The use of fossil-powered solution at mobile telecommunication sites presents a number of economic, logistical, and environmental problems [74]. The operation and maintenance of fossil fuelled generators account for about 78% of the total cost of operations (equivalent to about 35% of the total cost of ownership) of the GSM base transceiver station (BTS) sites [75]. In addition, [6, 9, 11] indicate that the earth's climatic change is the result of increasing concentrations of greenhouse gases resulting primarily from fossil fuel combustion into the atmosphere, yet Nigeria's grid electricity supply is characterized by high unreliability index [76]. Besides, the current and future demand patterns of energy are not sustainable [1]. Sustainable energy provides accessible, affordable, and reliable energy services that improve the socioeconomic and environmental standards within the overall developmental context of the society while recognizing equitable distribution [77]. This calls for an enabling technology that will integrate the renewable energy system with the national grid for a sustainable electricity supply to mobile telecommunication sites.

## 3. Proposed System Configuration

**Figure 3:**
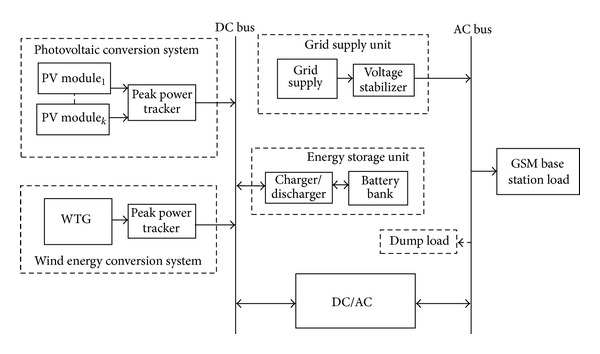
Block diagram of the proposed system structure for electricity supply.

Figure 3 shows the proposed hybrid system configuration. It consists of three primary energy sources (grid supply unit, wind energy conversion system unit, and photovoltaic conversion system unit), a battery bank, and a power conversion unit. Solar and wind energy systems are considered because they are both technically and environmentally viable options while on the other hand, the utility grid is the current source of electricity in Nigeria. The dump load consumes the excess energy generated by the system for proper functioning of the power conversion unit. The sustainability of the available energy resources within the region has informed the choice of the proposed system structure. Details of the model development and the optimal control strategy of the proposed system are available in [78, 79].

### 3.1. Problem Formulation

The objective is to minimize the proposed system's cost of energy (COE) in $/kWh defined by

(1) $$\begin{array}{c} \text{COE}=\frac{C_{\text{ann},\text{sys}}}{E_{\text{ann},\text{sys}}} \end{array}$$

and subject to the reliability condition

(2) $$\begin{array}{c} S_{i,\min}\leq S_{i\;}\leq S_{i,\max},\\ \text{LPSP}=0, \end{array}$$

where *S*_*i*_ is the size of each system's component *i*, *S*_*i*,min⁡_ and *S*_*i*,max⁡  _ are the minimum and maximum acceptable values for *S*_*i*_, LPSP is the loss of power supply probability of the system, and *E*_ann,sys_ is the total annual energy demand to be served by the system (kWh/yr). The system's annualized cost (*C*_ann,sys_) is defined by

(3) $$\begin{array}{c} C_{\text{ann},\text{sys}}=\frac{1}{L_{N}}\overset{i}{\underset{i=1}{\sum }}\left(C_{i,\text{ini}}+C_{i,\text{rep}(\text{PW})}+C_{i,\text{om}(\text{PW})}-C_{i,\text{sal}(\text{PW})}\right), \end{array}$$

where *C*_ini_ is the total initial cost, *C*_rep(PW)_ is the total present worth (PW) of replacement cost, *C*_OM(PW)_ is the total PW of annual operation and maintenance (OM) cost, *C*_sal(PW)_ is the PW of all salvage value, *L*_*N*_ is the lifespan of the project (yr), and *i* is the total number of the system's component unit with all cost expressed in $. An LPSP of zero is chosen with the aim of achieving 100% power supply reliability. Detailed analysis of (3) is available in [34].

### 3.2. Methodology

This study adopts the methodology proposed in [78, 79] to ensure optimal power generation among the three energy sources and for the effective and reliable distribution to satisfy the energy demand of GSM base station sites in emerging countries. The overall process control applies the genetic algorithm-based techniques for optimal sizing of system components and constrains the power generated and the distribution to reliably satisfy the energy demand while ensuring the safe operation of the system. At any time *t*, the sizing algorithm looks into the process of selecting the best combination of resources with proper operation strategy to provide efficient, cost-effective, constant, and reliable energy supply, and the best possible model is one that satisfies the user constraints at the lowest net present cost. Detailed description of the optimal control strategy is available in [78, 79].

The study develops a simulation model for the studied system structure in MATLAB and considers six locations (Katsina, Yola, Abuja, Nsukka, Lagos, and Benin City), representing the six geopolitical zones. These locations cut across the different climatic conditions in Nigeria. A comprehensive description of the climatic conditions of these locations is available in [12]. The performance validation of the proposed approach and the comparison with the current scenario applied data discussed below, and assessed future scenario till the year 2020, on the assumption of future population and mobile infrastructure growth rates of 2.5 and 25% per annual, respectively. The annual assumed interest, inflation, and escalation rates are 8, 10, and 6%, respectively. An overall cost of the grid purchased electricity of about $0.1519/kWh (including tax, meter maintenance, and connection fees) is assumed in this study.

#### 3.2.1. Meteorological and Load Data

The climatic condition in a given geographical region influences the renewable energy; hence, it is site/location dependent. Nigeria is located within the Equator and the Tropic of Cancer with main latitude and longitude of 10°N and 8°E, respectively. Although the latitude of Nigeria falls within the tropical zone, its climatic conditions are not entirely tropical in nature. There is a considerable amount of variation in the meteorological conditions of different regions of the country [78]. For example, the climatic condition of the north is arid, tropical at the centre, and equatorial in the southern part of the country.

**Figure 4:**
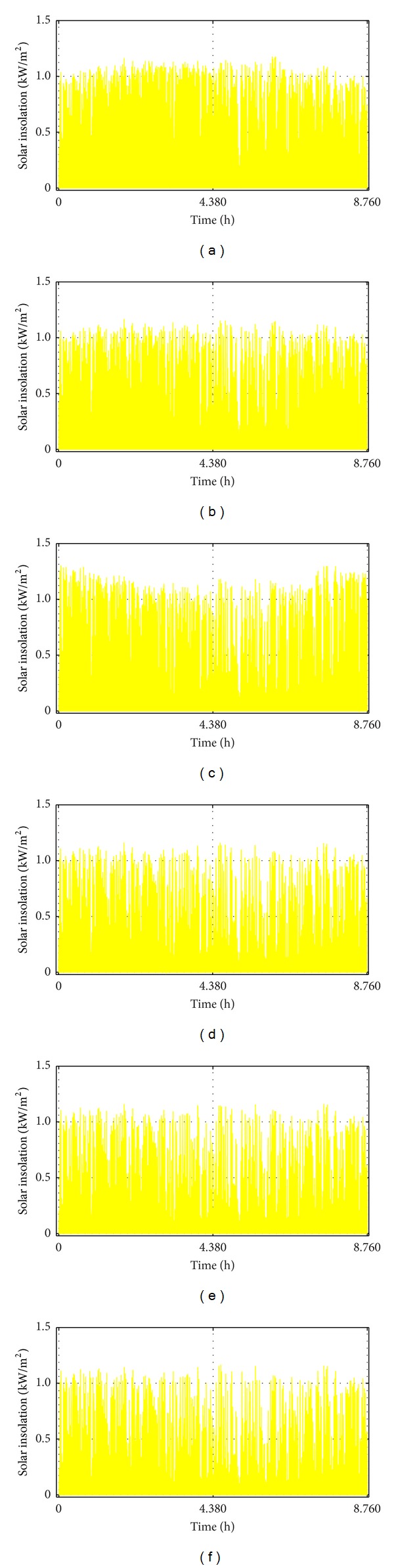
Computed hourly solar radiation on tilted PV surface using 22-year monthly average daily solar radiation data [16] for (a) Katsina, (b) Yola, (c) Abuja, (d) Nsukka, (e) Lagos, and (f) Benin City, respectively.

**Figure 5:**
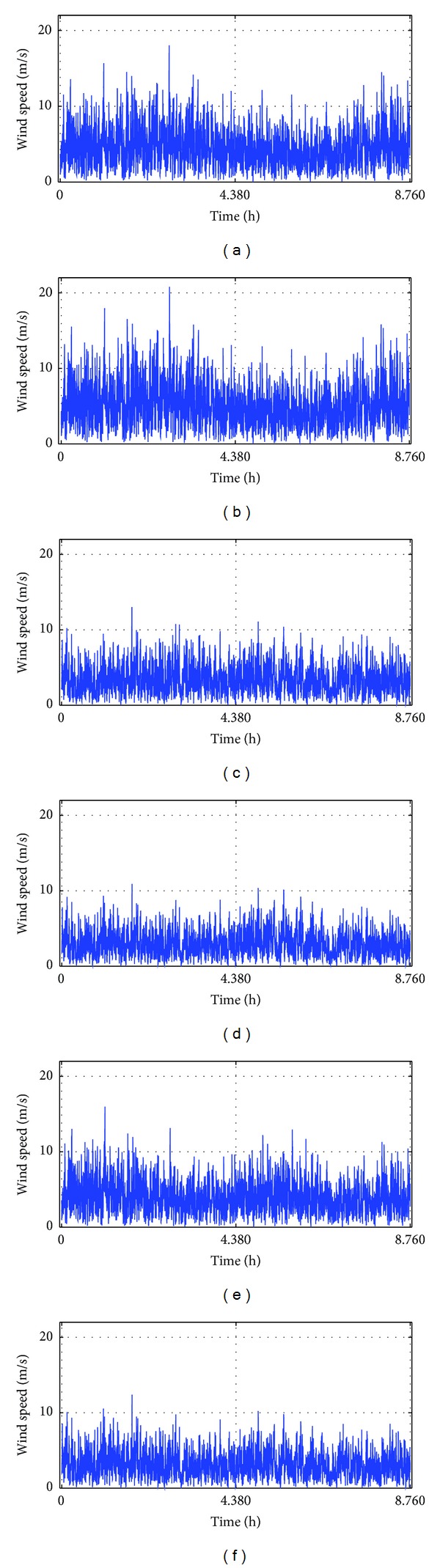
Computed hourly wind speed data using 10-year monthly average daily wind speed data [16] for (a) Katsina, (b) Yola, (c) Abuja, (d) Nsukka, (e) Lagos, and (f) Benin City, respectively.

In the absence of hourly data, this study applied the HOMER software [80] to convert the available monthly average daily datasets for the study locations to the hourly average datasets as shown in Figures 4 and 5, respectively. These datasets collected from the archives of the National Aeronautics and Space Administration (NASA) consist of 22-year (July, 1983–June, 2005) solar radiations on a horizontal surface and a 10-year (July 1983–June 1993) wind speed data at 100 m above the surface of the earth for flat rough grass surface. NASA derived these data from a variety of earth-observing satellites and reanalysis research programs with high accuracy [16]. Samples of daily hourly load profile collected for a typical GSM outdoor base station in Benin City were adjusted using the method discussed in [78] to account for the real time variations.

**Figure 6:**
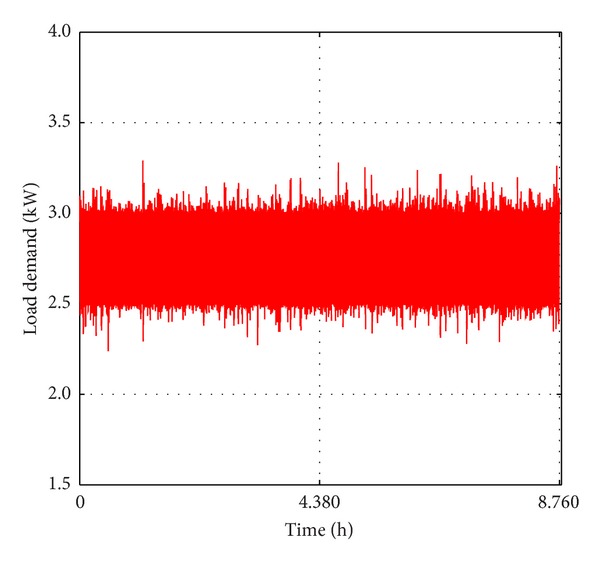
Adjusted hourly load profile for GSM outdoor base station.

Figure 6 shows the adjusted load profile for the GSM outdoor base station. For ease of comparison, the same load profile has been used in this study.

#### 3.2.2. Key Performance Indices

The energy contribution of the system expressed in terms of the percentage contribution of the individual sources is defined as

(4) $$\begin{array}{c} EC_{\text{es}}=\frac{100\times \eta_{\text{es}}\sum_{t=1}^{N}E_{\text{es}}\left(t\right)}{\sum_{t=1}^{N}E_{\text{hs}}\left(t\right)}, \end{array}$$

where *E*_es_ and *E*_hs_ are the energy production of each source (es) and the hybrid system, respectively, for a simulation period *N* (considered in this study as 8760 h).

The performance characteristic of the proposed system is determined in terms of its reliability in providing a cost-effective energy solution for satisfying the energy demand within the entire period *N*. The reliability of the energy supplied defined in terms of the ratio of the total deficit energy supplied to the total load required during the period *N* is

(5) $$\begin{array}{c} \eta_{\mathrm{rel}}=1-\text{LPSP}, \end{array}$$

and the loss of power supply probability (LPSP) is

(6) $$\begin{array}{c} \text{LPSP}=\frac{\sum_{t=1}^{N}E_{\mathrm{def}}\left(t\right)\;}{\sum_{t=1}^{N}E_{d}\left(t\right)}, \end{array}$$

where *E*_*def*_(*t*) and *E*_*d*_(*t*) are the deficit energy supplied and energy demand, respectively, at time *t*, both expressed in kWh. The energy throughput of the system is defined by [78] as

(7) $$\begin{array}{c} K_{e}=\frac{\eta_{\mathrm{rel}}}{\text{COE}}. \end{array}$$

Equation (7) is an expression of the technoeconomic viability of the system per unit cost of energy supplied. A higher energy throughput is an indication of a more superior system performance.

## 4. Results and Discussions

Table 1 shows the results of the proposed grid-connected hybrid PV-wind energy system, under the same load demand profile for (a simulation period of 1 year) the studied locations in Nigeria.

**Figure 7:**
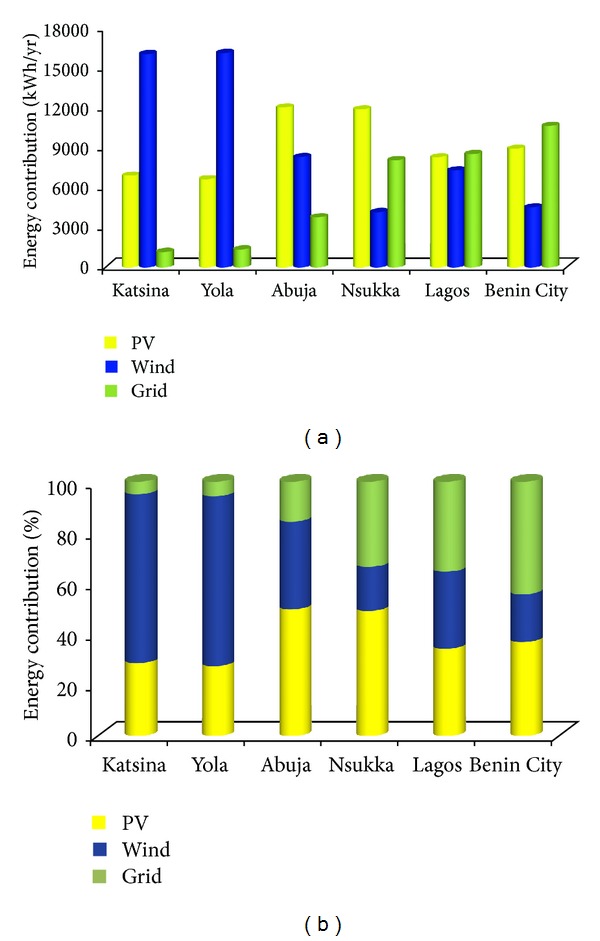
Electrical characteristic of proposed hybrid energy system: (a) energy contribution and (b) percentage contribution.

A thorough study of the system's key performance indices indicates that the renewable energy contribution increases with increasing latitudes; that is, the percentage of grid electricity purchased decreases from the southern to the northern parts of Nigeria, as shown in Figure 7. This is obviously owing to the dependance of renewable resources on climatic conditions. The larger percentage contribution of renewable sources throughout the studied locations is a reflection of the vast resource availability and the economic and technical viability of the renewable resources for a sustainable electricity supply to mobile telecommunication sites in Nigeria.

There is about 11% variation of the renewable energy contribution in the northern part of the country. This variation is compensated for through grid electricity purchased. Renewable energy contribution varies from about 84.3% in Abuja to about 94.4% and 95.1% in Yola and Katsina, respectively, as shown in Figure 7(b). By evaluating the wind speed data presented in Figure 4, it is clear that the operating hours of the WTGs in Katsina and Yola are larger compared to Abuja. This translates into a higher percentage of wind energy contribution (of about 67%) in Katsina and Yola and this is more than twice as high as the corresponding solar potential (28.6% and 27.5%) within the north-eastern and north-western Nigeria. At the centre, solar has a higher contribution of 50% compared to the 34.5% of wind energy, an indication of the densely populated Abuja city.

There is a significant variation of wind energy potential of about 50% to the total energy contribution within the country. The contribution of wind energy is largely limited by the sharp decrease in the annual average wind speed in the southern part of the country. The performance of the WECS varies only from about 18% in Benin City and Nsukka to 30.3% in Lagos. As a result, a larger fraction of solar energy is required to compensate for the available grid electricity supply. The increase in Lagos is an indication of the presence of the south-west trade wind that blows from the Atlantic Ocean in this region. The performance of solar conversion system is almost evenly distributed with peak values of about 50% at the centre (i.e., Abuja and Nsukka). Compared to the northern part of the country, the renewable sources contribute about two-third of the total energy requirement of base station sites.

**Figure 8:**
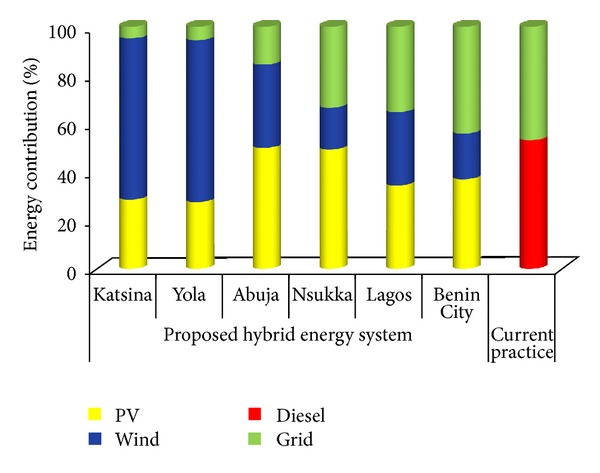
Comparison of electrical characteristics between the proposed system and the current traditional system.

A comparison of the proposed system's performance with the current practice of using the grid-diesel (diesel-fuelled generators as backups to electrical grids) system is shown in Figure 8. It indicates that the diesel generator is required to supply a minimum of about 53% of the total energy requirement of the mobile telecommunication base station sites. This corresponds to about 4526 hours of operating the diesel generator annually for the energy demand to be effectively satisfied. The implementation of the proposed system would eliminate the need for the fossil fuelled generators and reduce the dependence of the base station load on the erratic grid supply from about 47% presently to as low as about 4.9% in Katsina. This would in turn translate to a reduction in the pollutant emissions released into the atmosphere as a result of the consumption of electricity produced by the grid and fossil fuelled generators. To limit the scope of the study, only the technoeconomic performance of the proposed system has been considered.

**Figure 9:**
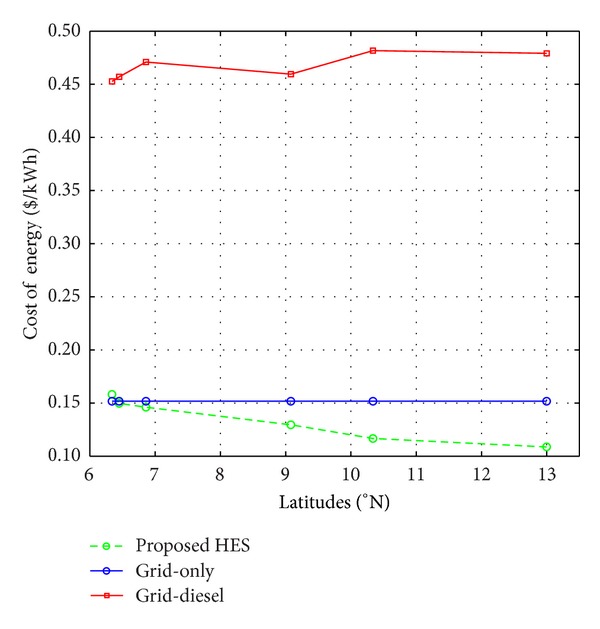
Variation of the economic cost of the proposed and existing energy systems with geographical locations.

By comparison with the current practise, the implementation of the proposed system indicates that an average performance improvement of over 200% could be achieved in the operation and maintenance cost of the GSM base station sites within the studied locations.  Figure 9 shows the comparison of the economic cost curves of the proposed, grid-diesel, and the grid-only (standard grid) energy systems within the studied locations. The variation in the cost of grid-diesel produced electricity results from the cost variation per litre of diesel consumed owing to additional cost of transportation from one part of the country to another.

**Figure 10:**
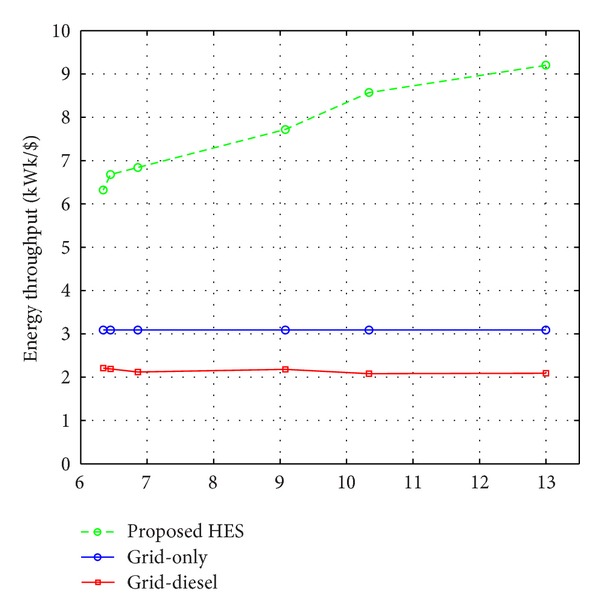
Variation of the energy throughput of the proposed and existing energy systems with geographical locations.

The larger percentage renewable contribution within the northern part of the country has significant improvement in the overall system cost of energy supplied by the proposed system. In Benin City (lat. 6.34), the grid-only energy supply system tends to provide a higher economic performance of about 5% over the proposed system but at a technical cost of about 53%, that is, at a power supply probability of about 47%. Conversely, the energy throughput of the grid-only energy supply system is lower, 3.09 kWh/$ as compared to 6.32 kWh/$, as shown in Figure 10. This is an indication that the grid-only energy supply system is not sustainable in this region. As the percentage of grid electricity purchased decreases from southern to northern Nigeria, the cost of the energy supplied by the system decreases, with an improvement exceeding 200% in the north. The corresponding energy throughput varies from about 6.32 kWh/$ in Benin City to about 9.20 kWh/$ in Katsina with an average of about 7 kWh/$ within the country. The larger energy throughput of the proposed system compared to the existing technology is a justification of the technoeconomic viability of the wind and solar resources for a sustainable electricity supply to mobile telecommunication sites in the country. Figure 10 shows the variation of the energy throughput of the proposed and existing energy systems with geographical locations.

**Figure 11:**
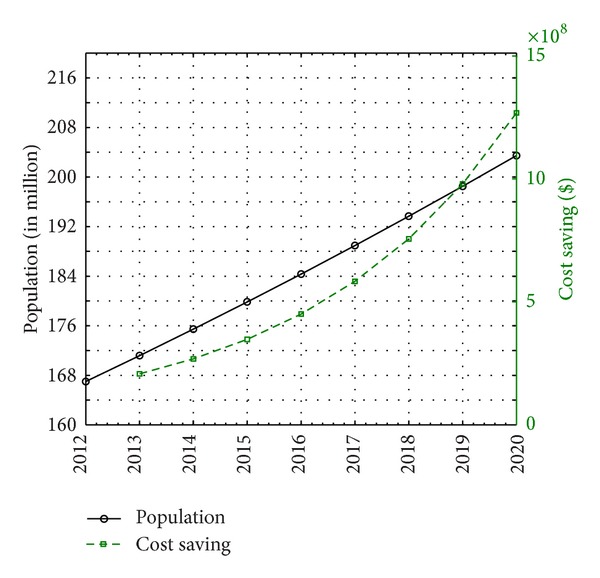
Comparison of the growth in cost saving and population till 2020.

The average energy throughput of the proposed grid-connected hybrid renewable energy system is equivalent to an annual cost of electricity consumption of about $65,040,000 (with $3,252 per site) required for running GSM BTS sites in Nigeria. This corresponds to an annual additional profit of about $207,210,000 at present and $4,861,600,000 by 2020 in the Nigerian mobile telecommunications sector owing to the improvement in the proposed system compared to the current scenario of the grid-diesel energy system. With the investment of the gains in infrastructural development, an additional increase exceeding 6% in the annual infrastructural growth rate could be achieved. Given a projected number of 120,000 GSM BTS sites in the country by 2020, the corresponding number of subscribers per cell site should be in the neighbourhood of about 1700 as this figure is expected to improve the quality of service delivery of the Nigerian mobile telecommunications sector based on global best practise. The projection is in line with the country's target (Vision 2020) of becoming one of the twenty world's leading economy in the year 2020. With an overall gain of over $4.86 billion (equivalent to about 768.13 billion naira), the Nigerian mobile telecommunications sector would be better positioned towards fulfilling its social obligations to the host communities.  Figure 11 shows the comparison of the growth in cost saving and population till 2020.

The results presented in Table 1 also indicate that the implementation of the stand-alone PV-wind hybrid energy system could be a more viable option. Conversely, the limited land area of most cell sites may constitute a major bottleneck on its feasibility on already existing sites in the country. Although remote cell site expansion may be feasible, the possibility of expansion in most cities in the country is not always possible. As the country is about to witness a tremendous mobile infrastructural development in the coming years, mobile operators should consider the green-mobile infrastructure. This would reduce the larger land area needed for the larger optimal operational sizes of the stand-alone hybrid renewable energy system. Notwithstanding, a critical analysis on the technoeconomic viability and the control strategy for the stand-alone system is essential, and this is currently underway.

The government, on the other hand, through relevant agencies and legislation should overhaul the telecommunications sector and develop policies that will encourage extensive utilization of energy efficiency and conservation methods. This should include the use of green-mobile base stations, that is, installing energy efficient infrastructure at new sites and the gradual replacement of an inefficient conversion process with an efficient one. The policy framework should specify the modality for the replacement of all inefficient conversion processes with efficient ones. In addition, there should be regulatory policies that will speed up the implementation of policy guidelines to enable the enforcement of the relevant environmental protection laws that will enhance sustainable development in the country. Notwithstanding, adequate funding is essential for motivating renewable and sustainable energy research in the country.

## 5. Conclusion 

This study reviewed the underlying principles of the RET in sufficient details and proposed a grid-connected hybrid PV-wind energy system incorporating a power conversion and energy storage unit. The comprehensive analysis on key performance indicators of the proposed system and the proposal of enabling legislation to facilitate implementation would inform decision making on the use of renewable energy resources on a much larger scale than presently in virtually all sectors of the economy. The extensive utilization of the renewable energy resources and energy efficient infrastructure would not only improve the quality of GSM mobile services in Nigeria, by making the operation of GSM BTS sites more cost effective, but also improve the living standard of the citizenry.

## Figures and Tables

**Table 1:** Simulation results of the proposed system for studied locations.

Region	Cities	Locations		Key performance indices (KPI)				
		Lat. (°N)	Long. (°E)	Energy contribution (%)			*K*_e_ (kWh/$)	Cost ($/kWh)
				PV	Wind	Grid		
NW	Katsina	13.00	7.60	28.63	66.49	4.88	9.20	0.109
NE	Yola	10.38	12.87	30.58	62.71	6.71	8.57	0.117
NC	Abuja	9.08	7.53	51.84	34.84	13.32	7.72	0.130
SE	Nsukka	6.86	7.39	51.32	18.01	30.68	6.84	0.146
SW	Lagos	6.45	3.40	35.12	30.92	33.96	6.68	0.150
SS	Benin City	6.34	5.63	37.02	18.81	44.17	6.32	0.158

NW: north-western, NE: north-eastern, NC: north-central, S: south-eastern, SW: south-western, and SS: south-south.
